# Lipoprotein(a) and diet—a challenge for a role of saturated fat in cardiovascular disease risk reduction?

**DOI:** 10.1016/j.ajcnut.2023.05.017

**Published:** 2023-05-11

**Authors:** Hayley G. Law, Frederick J. Meyers, Lars Berglund, Byambaa Enkhmaa

**Affiliations:** 1 Department of Internal Medicine, School of Medicine, University of California Davis, Sacramento, CA, United States; 2 Center for Precision Medicine and Data Sciences, School of Medicine, University of California Davis, Sacramento, CA, United States

## Abstract

In this perspective, we discuss new evidence relating to current dietary recommendations to reduce SFA intake to modulate an individual’s global risk of CVD. Although it is well established that lowering dietary SFA intake has a beneficial effect on LDL cholesterol concentrations, findings increasingly indicate an opposite effect on lipoprotein(a) [Lp(a)] concentrations. In recent years, many studies have firmly established a role for an elevated Lp(a) concentration as a genetically regulated, causal, and prevalent risk factor for CVD. However, there is less awareness of the effect of dietary SFA intake on Lp(a) concentrations. This study discusses this issue and highlights the contrasting effect of reducing dietary SFA intake on LDL cholesterol and Lp(a), 2 highly atherogenic lipoproteins. This calls attention to the need for precision nutrition approaches that move beyond a “one-size-fits-all” approach. To illustrate the contrast, we describe the dynamic contributions of Lp(a) and LDL cholesterol concentrations to CVD risk during interventions with a low-SFA diet, with the hope that this will stimulate further studies and discussions regarding dietary management of CVD risk.

The role of dietary modification in reducing cardiovascular-related morbidity and mortality has been a focus of studies for many years, going back to the Seven Countries Study [1]. Although reduction of the intake of SFA has been robustly associated with lower LDL cholesterol concentrations and a decreased CVD risk, there has been more uncertainty regarding optimal replacement strategy for SFA [2]. However, given the consistent lowering effect of LDL cholesterol by reducing SFA intake, recent observations that this same intervention strategy resulted in an increase in lipoprotein(a) [Lp(a)] concentrations are noteworthy [3]. Lp(a) was initially identified by Berg [4] as a factor present in an LDL fraction used to immunize rabbits around the time when diet modification was shown to affect CVD risk. The discovery of the molecular structure, using protein sequencing and cDNA cloning, of apo(a)—the unique structural component of Lp(a)—identified the homology of apo(a) to plasminogen [5,6]. This led to improvements in Lp(a) assessment methods and the characterization of the importance of apo(a) size variability as a major genetic regulator of Lp(a) concentrations [7,8]. Elevated Lp(a) concentrations identified individuals at high risk of CVD. More recent studies identified molecular components of Lp(a) as having potential atherogenic importance, such as oxidized phospholipids, lipoprotein-associated phospholipase A_2_, and proprotein convertase subtilisin/kexin type 9 [7,9,10]. Targeted interventions to lower Lp(a) concentrations, and their effect on CVD risk, are currently being tested in clinical studies [7,11,12]. Evidence also suggests a role for Lp(a) in aortic valve calcification and diabetes mellitus [11,13]. Thus, ∼60 y after its discovery, an abundance of studies have shown that Lp(a) is a causal, independent, and prevalent risk factor for atherosclerotic CVD [14]. Recently, multiple guidelines have recommended intervention strategies to reduce the risk associated with elevated Lp(a) concentrations [11,15].

Although it is well established that Lp(a) concentrations are primarily genetically regulated, some nongenetic factors have been identified as predictors of plasma concentrations [16]. Thoroughly identifying such predictors has been a challenging task because multiple factors, including but not limited to sample size, choice of study design, genetic variability, and the choice of assays, have varied across studies. However, a few conditions have emerged that influence Lp(a) concentrations [17]. These can be broadly classified into 2 categories based on the direction of effect: *1*) Lp(a) increasing, and *2*) Lp(a) decreasing (Figure 1). Those factors that correlate with an increase in Lp(a) concentration include reduction in dietary SFA intake, hypothyroidism, menopause, growth hormones, chronic kidney disease (CKD), and dialysis treatments [17]. Those factors that correlate with a decrease in Lp(a) concentration include a diet low in carbohydrate and high in SFA, hyperthyroidism, hormone replacement therapy, and certain liver diseases [17]. The quantitative impact on Lp(a) at a population level by these metabolic and environmental factors is relatively less than the impact of genetic regulation of expression. Despite this, the impact can be substantial under some conditions.

**FIGURE 1:**
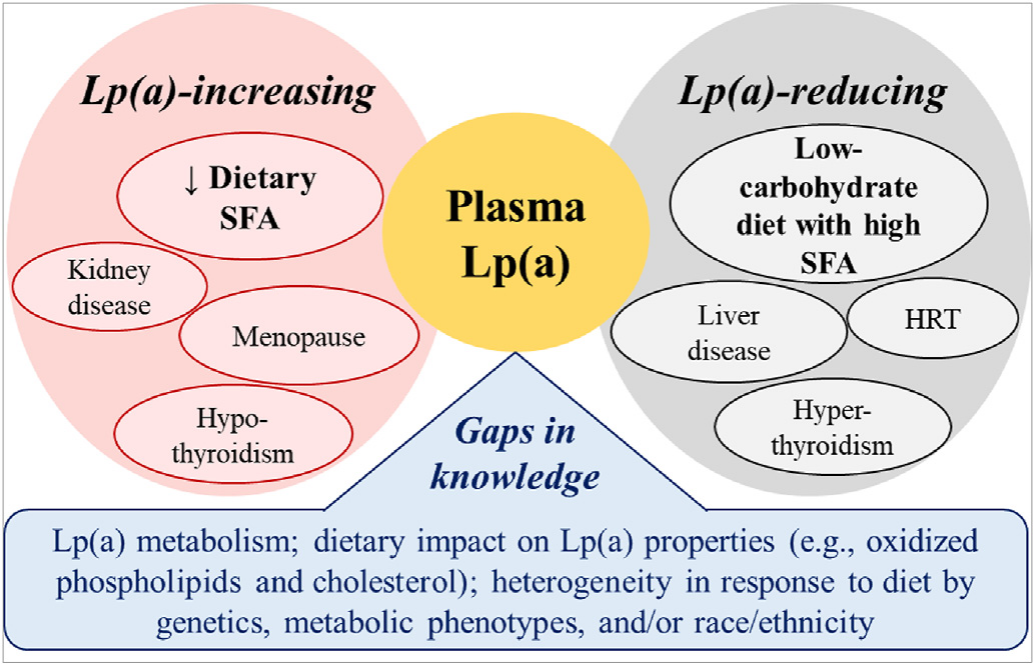
Examples of nongenetic factors influencing Lp(a) concentrations and knowledge gaps. HRT, hormone replacement therapy; Lp(a), Lipoprotein(a).

The effect of diet, and particularly reduction in dietary SFA, on Lp(a) concentration has been the subject of many studies. From the first report in 1991 [18], evidence generated in the 3 subsequent decades has provided robust support for a role of dietary regulation of Lp(a) concentrations [3,17,19]. Most available evidence to date strongly suggests that replacement of dietary SFA with other macronutrients—such as MUFA or carbohydrates—increases Lp(a) concentrations whereas consistently decreasing LDL cholesterol concentrations [3,20]. A recent study in a large number of persons of African descent confirmed this observation because dietary SFA reduction and replacement with primarily carbohydrates resulted in a 24% increase in Lp(a) concentrations and a concurrent decrease in LDL cholesterol by ∼10% [21]. Other studies have observed an increase in Lp(a) concentrations, and decreases in LDL cholesterol, after adoption of a Mediterranean-style diet replacing SFA with primarily MUFA [22]. Although the clinical significance of this discordance in Lp(a) and LDL cholesterol responsiveness is not well understood, the opposite effect of the SFA reduction on these 2 highly atherogenic lipoproteins raises some questions regarding dietary recommendations. Although it is premature to draw any definitive conclusions, it seems likely that any net effect on CVD prevention might be affected by the relative balance between LDL cholesterol and Lp(a) concentrations in a given individual. On the basis of current guidelines for prevention and management of dyslipidemia to reduce CVD risk, an individual with an elevated LDL cholesterol would be advised to replace dietary SFA with unsaturated fat [15,23,24]. With a low-SFA diet, LDL cholesterol concentrations would decrease significantly (7%–11%); however, Lp(a) concentrations would simultaneously increase by ≤24%. Depending on the individual’s baseline Lp(a) (high compared with low) and LDL cholesterol (moderate compared with high) concentrations, the relative effect of these 2 factors regarding CVD risk may vary. In general, a reduction in LDL cholesterol remains an important goal for risk reduction and reduction in SFA intake (e.g., with a Mediterranean-style diet) has been shown to be an important part of such a strategy [25,26]. However, clinical measurements of LDL cholesterol commonly include cholesterol contained in Lp(a) [Lp(a) cholesterol], typically estimated at 30% of Lp(a) mass [14]. Although this estimate has been widely used to correct LDL cholesterol for the contribution of Lp(a) cholesterol, a recent study reported a significant interindividual heterogeneity in the Lp(a) cholesterol content (6%–57%), on average, considerably less than the historical 30% value [27]. This variability raises concerns regarding the interpretation of the LDL cholesterol response. In particular, in individuals with elevated Lp(a) concentrations, the contribution of Lp(a) cholesterol to LDL cholesterol can be substantial, resulting in a notable difference in RR based on intervention studies [27]. Hence, precision CVD risk assessment and management will require measurement of LDL cholesterol that is not confounded by Lp(a) content, or, alternatively, a direct measurement of Lp(a) cholesterol. Such precision-guided assessments will be important to accurately quantify the actual reduction in LDL cholesterol concentration in the context of increased Lp(a) concentrations during dietary interventions. The question arises whether the differential effect of saturated fat compared with carbohydrate intake on LDL properties might contribute to the findings. Thus, although dietary carbohydrates are associated with increased de novo lipogenesis and increased concentrations of small, dense LDL particles, the lower LDL cholesterol concentration in response to dietary SFA reduction primarily reflects lower concentrations of large LDL particles [28]. Because Lp(a) particles reflect LDL properties, such as density and size [29], one could speculate that the increased Lp(a) concentrations associated with a decrease in saturated fat intake might also include potentially atherogenic changes in its lipid composition. These observations further support the notion that the effect of dietary changes on CVD risk cannot be reliably reflected by changes in LDL cholesterol concentrations alone and warrant additional biomarkers [e.g., Lp(a)] for assessing CVD risk and monitoring diet-induced effects in research and clinical practice.

The need for a better estimation of dietary effect is well illustrated in CKD. Many studies have consistently demonstrated an increase in Lp(a) concentrations in parallel with a reduction in kidney function, leading to a 3-fold elevated Lp(a) concentration in patients with advanced stages of CKD [17,30]. Patients with CKD (stages 1–5 not on dialysis or posttransplantation) with or without dyslipidemia are advised to follow a Mediterranean-style diet to improve their lipid profile [31]. The effect of this recommendation on Lp(a) and its associated atherogenic properties in CKD remains understudied. Further studies are clearly needed to address the potential synergetic effect on Lp(a) by CKD and diets low in SFA and their associations with CVD risk.

Although studies to date support a role for Lp(a) as a CVD risk factor, there is more uncertainty regarding a causal role of reducing Lp(a) concentrations in CVD risk reduction. However, it seems likely that an increase in Lp(a) concentration because of SFA reduction might play an underappreciated role as a CVD risk determinant in individuals with very high Lp(a) concentrations and minor-to-modestly elevated LDL cholesterol concentrations. Given the high prevalence of elevated Lp(a) (∼20%–25% of the population, i.e., ∼1.4 billion people worldwide) [32], these scenarios highlight the importance of precision nutrition approaches that move beyond a “one-size-fits-all” dietary prescription for optimal health and disease prevention [33]. Knowledge gaps remain regarding Lp(a) properties, such as phenotypic characteristics, affecting atherogenicity and its largely unknown metabolism (Figure 1). Therefore, a more detailed characterization of the effect of dietary changes on atherogenic properties of Lp(a), such as the contents of oxidized phospholipids and cholesterol, is needed. Furthermore, a greater understanding of the heterogeneity in Lp(a) responsiveness to diet owing to genetics, e.g., variation due to apo(a) size polymorphism, race/ethnicity, and metabolic phenotype, is required. Because current evidence in these areas is limited, studies that address these existing knowledge gaps will help identify individuals who would benefit the most (or the least) from the generalized dietary recommendations (Figure 1). Such efforts would strengthen the foundation for individualization—a necessary effort in the era of precision medicine.

Taken together, at present, insufficient evidence exists to make dietary recommendations for patients with elevated Lp(a) concentrations. Evidence from well-controlled clinical investigations suggests that diet—particularly a reduction in SFA intake—affects Lp(a) concentrations, often in the opposite direction of LDL cholesterol concentrations. Dietary changes, as a common nonpharmacologic therapy, are critical for the prevention and management of CVD risk at the level of public health. To maximize the beneficial effect, dietary recommendations should be individualized whenever possible. In this context, more research is needed to address the potential challenge of dietary saturated fat in CVD risk reduction in diverse patient and population groups.
